# Supplementary material for: Pediatric overweight and obesity increased in Israel during the COVID-19 period
Source: PLoS One. 2023 Sep 5;18(9):e0290961. doi: 10.1371/journal.pone.0290961 (PMC10479915; doi:10.1371/journal.pone.0290961)
Supplement: S1 Checklist — (DOCX) [file pone.0290961.s001.docx]

STROBE Statement—checklist of items that should be included in reports of observational studies

|  | Item No. | Recommendation | Page  No. | Relevant text from manuscript |
| --- | --- | --- | --- | --- |
| **Title and abstract** | 1 | (*a*) Indicate the study’s design with a commonly used term in the title or the abstract | 3 | Here, we present results of a longitudinal study of the entire population of Israel |
|  |  | (*b*) Provide in the abstract an informative and balanced summary of what was done and what was found | 3 | See abstract |
| Introduction | | | |  |
| Background/rationale | 2 | Explain the scientific background and rationale for the investigation being reported | 4-5 | See background section |
| Objectives | 3 | State specific objectives, including any prespecified hypotheses | 5 | Our main study questions were: did we see an increase in pediatric overweight and obesity in 2020 and 2021 compared to previous years, and were these changes worse among children of lower SES? |
| Methods | | | |  |
| Study design | 4 | Present key elements of study design early in the paper | 6 | 2.2. Dataset  QICH data are organized by calendar year. Therefore, in this manuscript, we will be comparing the years 2020 and 2021, coinciding with the COVID-19 pandemic, with the years 2017-2019, which were prior to the pandemic. The analyses reported here were conducted between September 2022-May 2023.  2.3. Measures  This manuscript will focus on two measures, which are part of the QICH quality measure set for Israel. The first measure is the proportion of children who have height and weight recorded, sufficient to calculate body mass index (BMI). Children are assessed for weight and height (in order to calculate BMI) at the age of 7 and at the age of 14-15. These assessments are carried out by a nurse or a physician at regular office visit.  The second measure Is the proportion of children who are in different weight categories. For this measure, the denominator consists of those children who have been assessed for both weight and height. The BMI categories are calculated according to the WHO growth charts… |
| Setting | 5 | Describe the setting, locations, and relevant dates, including periods of recruitment, exposure, follow-up, and data collection | 6 | Therefore, in this manuscript, we will be comparing the years 2020 and 2021, coinciding with the COVID-19 pandemic, with the years 2017-2019, which were prior to the pandemic. |
| Participants | 6 | (*a*) *Cohort study*—Give the eligibility criteria, and the sources and methods of selection of participants. Describe methods of follow-up  *Case-control study*—Give the eligibility criteria, and the sources and methods of case ascertainment and control selection. Give the rationale for the choice of cases and controls  *Cross-sectional study*—Give the eligibility criteria, and the sources and methods of selection of participants | 5 | In Israel, all citizens and permanent residents are members of one of the four health maintenance organizations (HMOs) that supply health services in the community [21]. All HMOs support and cooperate with QICH program, in development, assessment, providing the national indicators, data, and publication of the quality indicators. The data represent the entire Israeli civilian population. QICH data are aggregated by sex, age and socioeconomic status, and therefore they are de-identified and cannot be re-identified. These data are used for quality assurance purposes, and are the basis of annual public reports. QICH data are organized by calendar year. Therefore, in this manuscript, we will be comparing the years 2020 and 2021, coinciding with the COVID-19 pandemic, with the years 2017-2019, which were prior to the pandemic. |
|  |  | (*b*) *Cohort study*—For matched studies, give matching criteria and number of exposed and unexposed  *Case-control study*—For matched studies, give matching criteria and the number of controls per case |  | Not applicable – no matching |
| Variables | 7 | Clearly define all outcomes, exposures, predictors, potential confounders, and effect modifiers. Give diagnostic criteria, if applicable | 7 | Covariates included age (age 7 or age 14-15), sex, and area-level socioeconomic status (SES), as reported by the POINTS company [23]. POINTS divides Israeli residential locations into 10 levels of SES; we reduced those to 4 levels here, by combining levels 1-3 (very poor), 4-5 (moderately poor), 6-7 (middle income), and 8-10 (wealthy).  2.5. Analyses  We examined both measures by year, namely 1) the proportion of children who had a BMI measurement, and 2) the proportion of those measured who were in different BMI categories. We report these measures separately for age 7 and for age 14-15. We also stratified the population into SES groups to compare time trends across these groups, and present the rate ratio (RR) for the difference between lowest and highest SES. In addition, we present the data stratified by all three covariates (age, sex, and SES). We calculated a 99% Confidence Interval (99% CI) for all point estimates and rate ratios, as well as p-values for linear trend over the period from 2017-2021. All analyses were performed using R studio v 2022.07.2+576 and Microsoft Excel. |
| Data sources/ measurement | 8* | For each variable of interest, give sources of data and details of methods of assessment (measurement). Describe comparability of assessment methods if there is more than one group | *7* | We examined both measures by year, namely 1) the proportion of children who had a BMI measurement, and 2) the proportion of those measured who were in different BMI categories. We report these measures separately for age 7 and for age 14-15. We report these measures separately for age 7 and for age 14-15. We also stratified the population into SES groups to compare time trends across these groups, and present the rate ratio (RR) for the difference between lowest and highest SES. In addition, we present the data stratified by all three covariates (age, sex, and SES). We calculated a 99% Confidence Interval (99% CI) for all point estimates and rate ratios, as well as p-values for linear trend over the period from 2017-2021. |
| Bias | 9 | Describe any efforts to address potential sources of bias |  | Not applicable |
| Study size | 10 | Explain how the study size was arrived at | 5 | The data represent the entire Israeli civilian population. |

Continued on next page

| Quantitative variables | 11 | Explain how quantitative variables were handled in the analyses. If applicable, describe which groupings were chosen and why | 7 | We examined both measures by year, namely 1) the proportion of children who had a BMI measurement, and 2) the proportion of those measured who were in different BMI categories. We report these measures separately for age 7 and for age 14-15. |
| --- | --- | --- | --- | --- |
| Statistical methods | 12 | (*a*) Describe all statistical methods, including those used to control for confounding |  |  |
|  |  | (*b*) Describe any methods used to examine subgroups and interactions |  |  |
|  |  | (*c*) Explain how missing data were addressed |  | Not possible to ascertain missingness as these are de-identified data |
|  |  | (*d*) *Cohort study*—If applicable, explain how loss to follow-up was addressed  *Case-control study*—If applicable, explain how matching of cases and controls was addressed  *Cross-sectional study*—If applicable, describe analytical methods taking account of sampling strategy |  | Not possible to address loss to follow up as these are de-identified data |
|  |  | (*e*) Describe any sensitivity analyses |  | n/a |
| Results | | | | |
| Participants | 13* | (a) Report numbers of individuals at each stage of study—eg numbers potentially eligible, examined for eligibility, confirmed eligible, included in the study, completing follow-up, and analysed |  | Not applicable – data are delivered to us de-identified |
|  |  | (b) Give reasons for non-participation at each stage |  |  |
|  |  | (c) Consider use of a flow diagram |  |  |
| Descriptive data | 14* | (a) Give characteristics of study participants (eg demographic, clinical, social) and information on exposures and potential confounders | 8 | Table 1 |
|  |  | (b) Indicate number of participants with missing data for each variable of interest |  | Table 1 |
|  |  | (c) *Cohort study*—Summarise follow-up time (eg, average and total amount) |  | Not applicable |
| Outcome data | 15* | *Cohort study*—Report numbers of outcome events or summary measures over time |  |  |
|  |  | *Case-control study—*Report numbers in each exposure category, or summary measures of exposure |  |  |
|  |  | *Cross-sectional study—*Report numbers of outcome events or summary measures | *9-15* | *Tables 2-5* |
| Main results | 16 | (*a*) Give unadjusted estimates and, if applicable, confounder-adjusted estimates and their precision (eg, 95% confidence interval). Make clear which confounders were adjusted for and why they were included |  | See tables. 99% confidence intervals are given. Results are unadjusted. |
|  |  | (*b*) Report category boundaries when continuous variables were categorized | 6 | The BMI categories are calculated according to the WHO growth charts [22], meaning that Israel’s children are assessed compared to the international data underlying the WHO curves:   - Children with Underweight (BMI ≤2.3 percentile, z-score below -2) - Children with Normal Weight (BMI between 2.3-85 percentile) - Children with Overweight (BMI between 85-97.7 percentile, z-score between 1-2) - Children with Obesity (BMI above 97.7 percentile, z-score above 2) |
|  |  | (*c*) If relevant, consider translating estimates of relative risk into absolute risk for a meaningful time period |  | Not applicable |

Continued on next page

| Other analyses | 17 | Report other analyses done—eg analyses of subgroups and interactions, and sensitivity analyses | 17 | Table 6 |
| --- | --- | --- | --- | --- |
| Discussion | | | | |
| Key results | 18 | Summarise key results with reference to study objectives | 18-19 | We examined whether the prevalence of pediatric overweight and obesity increased during the COVID-19 period, namely in 2020 and 2021, compared to the previous three years (2017-2019). In 2020 and 2021, we saw a decrease in BMI documentation at ages 7 and 14-15…We also saw increased obesity rates in both age groups specifically among boys, and especially among the 14–15-year-olds, which revered a previous trend of improvement among younger children and accelerated a trend of worsening obesity among the older children… Our SES and sex-stratified results are among the most interesting findings here. Specifically, rates of obesity, and socioeconomic disparities in obesity, were much worse among 14–15-year-old boys than among girls of the same age. Moreover, this socioeconomic disparity worsened among boys during the COVID period and actually improved among girls. |
| Limitations | 19 | Discuss limitations of the study, taking into account sources of potential bias or imprecision. Discuss both direction and magnitude of any potential bias | 20-21 | However, our study also has limitations. Our data are aggregate, and therefore, certain types of individual-level analyses were not possible with these data. For example, it would not be possible to perform sensitivity analyses with different cutoffs for the BMI categories using these data.  Another possible limitation is that the decreases in documentation that we observed during the COVID period could have contributed to the apparent worsening of overweight and obesity during the same period. If the children that physically attended visits were different from those who used telemedicine, this could have biased our estimates of obesity. In particular, it is possible that children of low SES, or their parents, lacked the computer equipment to conduct a virtual visit, or were less aware that it was available. However, our sex and SES-stratified results, presented in Table 7, do not support this idea. We saw that socioeconomic disparities in obesity among 14-15-year-olds worsened among boys, but improved among girls. It does not seem plausible that the families of girls had better access to computers and telemedicine than those of boys. Another point in support of these data being real is that the documentation of height and weight decreased much more markedly among 14-15-year-olds than among 7-year-olds, and yet similar increases in obesity were observed in both age groups. Therefore, the increased prevalence of overweight and obesity that we observed during 2020 and 2021 would appear to be real, and not an artifact due to differential changes in documentation. |
| Interpretation | 20 | Give a cautious overall interpretation of results considering objectives, limitations, multiplicity of analyses, results from similar studies, and other relevant evidence | 19-20 | There have been numerous recent reports about childhood obesity rates during the COVID period. These reports have used various methods and have originated from various locations [7,8,10,13,14,17]. Two related reports about children treated in Kaiser Permanente of Southern California found increasing rates of overweight and obesity in that population during the COVID period. In their study, BMI increased by 1.72 for Hispanic youths, and 1.70 for Black youths, and only by 1.16 for White youths [12,18]. A longitudinal study of children treated by three large health systems in Massachusetts also showed increasing rates of overweight and obesity, again, with greater increases in obesity among minority populations, especially Black and Hispanic children [19]. A large, annual web-based survey in South Korea, which relied on self-reported height and weight, also found an increasing rate of obesity over this period, increasing from 11.0% to 12.1% [9]. In a study that echoes our findings, a school-based program in Isparta Province, Turkey, showed that improvements in the rates of overweight and obesity that had occurred since 2005 were suddenly reversed, with 2021 numbers being higher than any they had previously observed [16]. A study relying on a large, common electronic medical record in the USA found that BMI increased very quickly during the early pandemic, but then stabilized or even improved slightly later in the pandemic [15]. A probabilistic annual national sample from Peru showed that the proportion of children with obesity had increased from 6.4% to 7.8% between 2019 and 2021 [11]. |
| Generalisability | 21 | Discuss the generalisability (external validity) of the study results | 20 | Our study had important strengths. While some previous studies have relied on convenience samples, such as querying patients treated in a particular health system, others reflect a probability-based sample of a national population. However, ours is the first study of which we are aware that directly reflects the population of an entire country. |
| Other information | |  | | |
| Funding | 22 | Give the source of funding and the role of the funders for the present study and, if applicable, for the original study on which the present article is based | 22 | The Quality Indicators in Community Healthcare project is funded by the Israel Institute for Health Policy Research. |

*Give information separately for cases and controls in case-control studies and, if applicable, for exposed and unexposed groups in cohort and cross-sectional studies.

**Note:** An Explanation and Elaboration article discusses each checklist item and gives methodological background and published examples of transparent reporting. The STROBE checklist is best used in conjunction with this article (freely available on the Web sites of PLoS Medicine at http://www.plosmedicine.org/, Annals of Internal Medicine at http://www.annals.org/, and Epidemiology at http://www.epidem.com/). Information on the STROBE Initiative is available at www.strobe-statement.org.
