# Supplementary material for: Pediatric overweight and obesity increased in Israel during the COVID-19 period
Source: PLoS One. 2023 Sep 5;18(9):e0290961. doi: 10.1371/journal.pone.0290961 (PMC10479915; doi:10.1371/journal.pone.0290961)
Supplement: Attachment — Submitted filename: Response to Reviewers PONE-D-23-13315_r1.docx [file pone.0290961.s002.docx]

Inge Roggen, MD PhD, Academic Editor	July 13, 2023

PONE-D-23-13315_r1

Dear Dr. Roggen,

Enclosed please find our revision of “Pediatric Overweight and Obesity Increased in Israel During the COVID-19 Period”, an Original Research Article submitted for consideration of publication. This study has not been published elsewhere, in whole or in part, nor is it under consideration for publication elsewhere. The Israel Quality Indicators in Community Healthcare (QICH) program is supported by funds from Israel’s Ministry of Health. The sponsor had no role in the design and conduct of the study; the collection, management, analysis, and interpretation of the data; or the preparation, review, and approval of the manuscript. We have no conflicts of interest to report. All authors have agreed with the submission and approved the final version.

We have responded to the reviewers’ comments and the manuscript is much improved. Below, the reviewers’ comments appear in italics and our responses follow. Thank you for considering this revised manuscript.

Adam J. Rose, MD MSc – Corresponding Author

School of Public Health

Hebrew University, Ein Kerem Campus

Jerusalem, Israel

Telephone: +972-55-500-1040

Email: [adamrose@bu.edu](mailto:adamrose@bu.edu)

*1. Please ensure that your manuscript meets PLOS ONE's style requirements, including those for file naming.*

We went over the Instructions for Authors carefully and made several minor changes to ensure that we are in compliance.

*2. We note that you have indicated that data from this study are available upon request. PLOS only allows data to be available upon request if there are legal or ethical restrictions on sharing data publicly. In your revised cover letter, please address the following prompts:*

*a) If there are ethical or legal restrictions on sharing a de-identified data set, please explain them in detail (e.g., data contain potentially sensitive information, data are owned by a third-party organization, etc.) and who has imposed them (e.g., an ethics committee). Please also provide contact information for a data access committee, ethics committee, or other institutional body to which data requests may be sent.*

*We will update your Data Availability statement on your behalf to reflect the information you provide.*

QICH data are collected by agreement from Israel’s four health maintenance organizations (HMOs). The use of these data is governed by the data sharing agreement that allows the data to be collected in one place. One of the stipulations of this data sharing agreement is that the data must remain on a particular encrypted server located at Hebrew University, and must be analyzed from that location, and cannot be shared with outside parties. It is possible for outside researchers to apply to use the data in conjunction with the QICH Steering Committee. Analyses would be conducted by QICH staff, and the results would be sent to the collaborators, because the data cannot leave the server. Requests to work with QICH data should be sent to the QICH Steering Committee at contact@israelhealthindicators.org.

*3. We note that you have included the phrase “data not shown” in your manuscript. Unfortunately, this does not meet our data sharing requirements. PLOS does not permit references to inaccessible data. We require that authors provide all relevant data within the paper, Supporting Information files, or in an acceptable, public repository. Please add a citation to support this phrase or upload the data that corresponds with these findings to a stable repository (such as Figshare or Dryad) and provide and URLs, DOIs, or accession numbers that may be used to access these data. Or, if the data are not a core part of the research being presented in your study, we ask that you remove the phrase that refers to these data.*

Thank you for pointing this out. We have decided that this is not a core part of the study, and have therefore removed the phrase.

*Reviewer #1: The manuscript fails to clarify:
1. The difference between census and survey*

Our manuscript does not describe a census or a survey. Rather, it represents a re-use of data collected during healthcare delivery. Because of the fact that all healthcare in Israel is delivered by four health maintenance organizations, these data represent the entire population of the country. We have clarified this point in the manuscript. For example, from the methods:

In the State of Israel, all citizens and permanent residents are members of one of the four health maintenance organizations (HMOs) that supply health services in the community. All HMOs support and cooperate with QICH program, in development, assessment, providing the national indicators, data, and publication of the quality indicators. The data represent the entire Israeli civilian population. QICH data are aggregated by sex, age and socioeconomic status, and therefore they are de-identified and cannot be re-identified. These data are used for quality assurance purposes, and are the basis of annual public reports.

*2. compares with the data before the occurrence of COVID 19*

The purpose of the study was to compare data from 2020 and 2021 (years impacted by COVID) with prior years (2017-2019, years not impacted by COVID). This is stated in the study objective, at the end of the background, and such comparisons between COVID and pre-COVID periods constitute nearly all of the results presented. It is clear, therefore, that this study is comparing what happened during COVID with what was happening in the years prior to COVID.

*3. Establishing research territory*

The territory controlled by the State of Israel is a matter of international controversy, and is not the main topic of our paper. There is a sovereign State of Israel, within internationally recognized boundaries. There are also territories disputed between Israel and the Palestinian Authority. When our paper states that we have included the entire population of Israel, we mean all the citizens and legal permanent residents of the State of Israel, not including people who reside in disputed territory.

We added the words “the State of” before the word “Israel” in the methods to clarify this issue, as follows (the underlined words are new): “In the State of Israel, *all citizens and permanent residents* are members of one of the four health maintenance organizations…”

We also added the following text to the discussion (the underlined text is new):

Another possible limitation is that the decreases in documentation that we observed during the COVID period could have contributed to the apparent worsening of overweight and obesity during the same period. Indeed, our dataset is representative of the entire population of the State of Israel in that all citizens and legal residents receive care from the four Israeli HMO’s; however, there were certainly some children in the population who did not see a physician during this time. For this reason, we present confidence intervals in this paper, because our sample, while extremely representative, may not include every single person living in the State of Israel.

*4.Is the factor related to obesity occur in just ONE year?*

It is important to emphasize that each year’s data represents a cross-section of the Israeli population during that year. As such, the 14-15 year olds that are sampled in a particular year are not the same ones as those sampled the previous year. Therefore, the trends that are seen are actually not the same children gaining weight. Rather, they represent a new cohort of children who have a higher BMI than last year’s cohort. Therefore, our data must be understood at the population level, and should not be understood in the context of individuals gaining weight over time. We added the following to the limitations section:

Finally, when interpreting this study, it is important to keep in mind that each year’s data represents a cross-section of the Israeli population, and that the same individuals are not tracked from year to year. This has to do with the mechanism of collecting the data, as explained above. As such, our results should not be interpreted as saying that a group of Israeli children, followed across the life course, were gaining weight rapidly from year to year. Although this may have happened, our data cannot show this. Rather, our data should be interpreted as showing that successive age cohorts during the COVID period had higher BMI each year than the year before.

*5. The language, grammar and complete construction of sentence and paragraphs*

We have gone over the manuscript carefully to ensure that the grammar, language, and paragraphs are appropriate. If there is a specific line that presents an issue, please point it out and we will be happy to correct it.

*6.You have used confidence interval. Hence, is that sampled or per the entire population?*

The reviewer raises an interesting point, which is that confidence intervals are usually used for a statistically representative sample of the population, and not for an entire population. Although the reviewer is correct, we think many papers such as ours would still present a confidence interval. One way to justify this is that while our database does represent all the people who received medical care in Israel, there may be a small number of people who did not see a doctor at all during that period of their lives. Therefore, our dataset can be understood as a statistical sample, and therefore a confidence interval is in order.

We have addressed this issue by adding the following to the limitations section (the underlined text is new):

Another possible limitation is that the decreases in documentation that we observed during the COVID period could have contributed to the apparent worsening of overweight and obesity during the same period. Indeed, our dataset is representative of the entire population of the State of Israel in that all citizens and legal residents receive care from the four Israeli HMO’s; however, there were certainly some children in the population who did not see a physician during this time. For this reason, we present confidence intervals in this paper, because our sample, while extremely representative, may not include every single person living in the State of Israel. Relatedly, differential inclusion in the dataset may have impacted our study. If the children that physically attended visits were different from those who used telemedicine, this could have biased our estimates of obesity.

*7. Appropriate presentation of the result, discussion, conclusion and recommendations as well as reference sections!*

We have gone over these sections carefully and have ensured that they are appropriately presented. If there is a specific line that presents an issue, please point it out and we will be happy to correct it.

*Reviewer #2: Globally, overweight and obesity are serious public healthcare concerns. The recent outbreak of Covid-19 has revealed vulnerabilities in most health systems and socioeconomic disparities among the world population. Thank you for your efforts in making this significant contribution to literature.*

Thank you for this appreciative comment.

*However, I have some minor remarks in your discussion section. "Our study had important strengths." This is an overstatement. Only a single strength has been stated. Where are the other strengths? Please state the other strengths.*

Thank you. We added a second study strength: “In addition, we used a dataset that allowed year-by-year comparisons across three pre-COVID years (2017-2019) and two years impacted by COVID (2020-2021).”

*"Another point in support of these data being real is that the documentation of height and weight decreased much more markedly among 14-15-year-olds than among 7-year-olds, and yet similar increases in obesity were observed in both age groups."
This statement may be construed as if there is doubt about the authenticity of the study data. Could you reconsider structuring this statement?*

Thank you for noticing this. We changed the text to read as follows: “Another point is that the documentation of height and weight decreased much more markedly among 14-15-year-olds than among 7-year-olds, and yet similar increases in obesity were observed in both age groups. Therefore, the increased prevalence of overweight and obesity that we observed during 2020 and 2021 would appear to be real changes, and not an artifact due to differential changes in documentation.”

The implication that our data are not authentic has been removed. We had meant to refer to the question of whether there were real changes over time, or as opposed to an artifact. Hopefully the revised language makes this clearer.
